# RNA sequencing of LX-2 cells treated with TGF-β1 identifies genes associated with hepatic stellate cell activation

**DOI:** 10.1007/s11033-021-06774-3

**Published:** 2021-10-14

**Authors:** Jack P. Carson, Mark W. Robinson, Grant A. Ramm, Geoffrey N. Gobert

**Affiliations:** 1 School of Biological Sciences, Queen’s University Belfast, 19 Chlorine Gardens, BT9 5DL Belfast, UK; 2 QIMR Berghofer Medical Research Institute, Royal Brisbane Hospital, Locked Bag 2000, QLD 4029 Brisbane, Australia; 3 Faculty of Medicine, The University of Queensland, Level 6, Oral Health Centre (Building), Herston Road, 4006 Herston, QLD Australia

**Keywords:** Hepatic stellate cell, LX-2, Transforming growth factor-β1, Fibrosis, Chronic liver disease.

## Abstract

**Background:**

Hepatic stellate cells (HSCs) are liver-resident myofibroblast precursors responsible for the production of collagen and maintenance of the hepatic extracellular matrix (ECM). As such, they are generally associated with fibrotic liver diseases. HSCs become “activated” in response to tissue damage or pathogen invasion, a process most commonly driven by transforming growth factor-β1 (TGF-β1). Despite this, the full extent of TGF-β1 signalling in these cells is poorly understood. Clarifying the range and diversity of this signalling will further improve our understanding of the process of HSC activation.

**Methods and results:**

RNA sequencing was used to quantitate the transcriptomic changes induced in LX-2 cells, an activated human HSC line, following TGF-b1 treatment. In total, 5,258 genes were found to be significantly differentially expressed with a false discovery rate cut-off of < 0.1. The topmost deregulated of these genes included those with no currently characterised role in either HSC activation or fibrotic processes, including CIITA and SERPINB2. *In silico* analysis revealed the prominent signalling pathways downstream of TGF-β1 in LX-2 cells.

**Conclusions:**

In this study, we describe the genes and signalling pathways significantly deregulated in LX-2 cells following TGF-β1 treatment. We identified several highly deregulated genes with no currently characterised role in HSC activation, which may represent novel mediators of fibrotic responses in HSCs or the liver macroenvironment. This work may be of use in the identification of new markers of liver fibrosis and could provide insight into prospective genes or pathways that might be targeted for the amelioration of fibrotic liver disease in the future.

## Introduction

Fibrosis can be defined as the excessive deposition of ECM proteins, particularly fibrillar collagens, within a tissue [1]. In the liver, ECM protein deposition is often provoked by injury or disease, where it assists tissue regeneration and limits the spread of harmful pathogens [2]. Despite these benefits, the development of fibrosis can often have pathological consequences; the accumulation of excessive amounts of ECM proteins can result in tissue congestion, which disrupts blood flow and compromises organ function [1, 3]. If the provoking agent persists, fibrosis can further develop into a chronic condition resulting in severe changes to the liver architecture and ultimately leading to cirrhosis, liver failure and death [1, 4]. Liver fibrosis is a common pathology of several diseases, including chronic hepatitis C virus (HCV) infection, alcoholic liver disease (ALD), non-alcoholic fatty liver disease (NAFLD)-derived non-alcoholic steatohepatitis (NASH) and some parasitic diseases (schistosomiasis).

HSCs are a population of myofibroblast precursors located within the space of Dissé in the liver sinusoids [5]. HSCs represent 5–8% of all liver cells [6] and store ~ 80% of the body’s total vitamin A. Upon receiving stimuli in response to either liver damage or disease, these normally quiescent storage cells undergo a process of transdifferentiation, or “activation”, into myofibroblasts (aHSCs) [5]. Following activation, HSCs lose their ability to store vitamin A, develop a broader ‘stretched’ cytoplasm supported by filaments of α-smooth muscle actin (ACTA2), and adopt roles involved in tissue regeneration and the immune response against invading pathogens [5, 7]. The primary role of myofibroblasts is the production of collagen and other ECM components, and as such aHSCs are the main cell population responsible for fibrogenesis in the liver [4].

HSCs are driven to activate in response to a wide variety of cellular and pathogen-derived stimuli. These stimuli can include cellular components such as growth factors, interleukins, reactive oxygen species or damage-associated molecular patterns; and proteins, DNA or lipopolysaccharide from pathogens [8]. The various mechanisms of HSC activation have been reviewed by Tsuchida and Friedman [8]. The type of response levied by HSCs is largely dependent on the specific activating stimulus [4, 8]. A major driver of both HSC activation and liver fibrogenesis is the cytokine TGF-β1 [9, 10]. This cytokine is almost ubiquitously expressed throughout mammalian tissues and is involved in a wide variety of critical physiological processes, including both immune and inflammatory responses, cell differentiation and tissue repair [10].

HSCs often play conflicting roles within the context of liver damage or disease. Their ability to produce ECM components makes HSCs critical in tissue regeneration and, due to the immunologically relevant cytokines and chemokines they produce, they are also important in the response against invading pathogens [5, 7]. However, their primary role of ECM protein synthesis also renders them responsible for the fibrosis, and fibrosis-related pathology and morbidity, associated with many chronic liver diseases [4].

TGF-β1 signalling in HSCs has yet to be explored in-depth at the transcriptomic level. Herein we will describe the application of RNA sequencing and *in silico* pathway analysis to identify the initial genes and signalling pathways that are most strongly deregulated by TGF-β1 treatment in LX-2 cells, an immortalised human HSC line that retains many important features of primary HSCs [11]. This work should improve the understanding of the transcriptional processes associated with HSC activation. Given the involvement of aHSCs in liver disease, these findings may provide new insights into the gene networks involved in fibrogenesis that could be exploited as fibrotic markers or as the targets of therapeutics.

## Materials and methods

### Cell culture

LX-2 cells (Merck Millipore, Burlington, USA), an immortalised human aHSC line [11], were maintained in Dulbecco’s modified eagle medium (DMEM, ThermoFisher Scientific, Waltham, USA) supplemented with 2% foetal bovine serum (FBS, Sigma-Aldrich, St. Louis, USA), 100 units/ml penicillin/streptomycin (ThermoFisher Scientific) and 4 mM L-glutamine (L-Glu, ThermoFisher Scientific) at 37 °C and 5% CO_2_. Upon reaching ~ 80% confluency, LX-2 cells were detached from the culture flask using 0.25% trypsin-EDTA solution (ThermoFisher Scientific) and re-seeded according to a split ratio of 1:3.

### Immunofluorescence

Cells were seeded in 48-well cell culture plates (ThermoFisher Scientific) at a density of ~10,000 cells per well, cultured in DMEM with supplements and treated with 2.5 ng/ml TGF-β1 [12] (InvivoGen, San Diego, USA) where appropriate for 72 h. Cells were then fixed and permealised in ice cold methanol for 5 min, washed three times in phosphate-buffered saline (PBS) for 5 min each and blocked in 5% bovine serum albumin (BSA, Sigma-Aldrich) in PBS (Sigma-Aldrich) for 30 min at room temperature. Cells were then incubated overnight at 4 °C in primary antibody (ACTA2, 1:250 dilution, Abcam ab5694, Cambridge, UK) diluted in 5% BSA in PBS. The following day the cells were washed three times in PBS for 5 min each, incubated with secondary antibody (goat anti-rabbit IgG H&L, Alexa Fluor® 488, 1:1000 dilution, Abcam ab150077) diluted in 5% BSA in PBS for 1 h at room temperature, washed three times again and then incubated with 4′,6-diamidino-2-phenylindole (DAPI) solution (1:1000 dilution in PBS) for 15 min at room temperature. The cells were washed for a final three times and lastly covered with 250 µl of clean PBS prior to imaging. Images were taken on a total internal reflection fluorescence microscope (Leica, Wetzlar, Germany) in standard fluorescent mode.

### RNA isolation

Cells were seeded in 6-well cell culture plates (ThermoFisher Scientific) at a density of ~100,000 cells per well and cultured in DMEM with supplements for 24 h. At this time, the media was removed, and the cells were gently rinsed 3 times with warm PBS. The cells were then serum starved overnight in serum-starvation media (DMEM supplemented with 0.1% FBS, 1 unit/ml penicillin/streptomycin and 4 mM L-Glu). The following morning, 2.5 ng/ml TGF-β1 [12] was added to the cells where appropriate. Cells were cultured for a further 24 h, after which the media was removed, and the cells were rinsed 3 times with cold PBS. Total RNA was isolated from the cells using the GenElute Mammalian Total RNA Miniprep Kit (Sigma–Aldrich). Genomic DNA was digested during this process using the On-Column DNase I Digestion Set (Sigma-Aldrich). RNA purity was assessed using the POLARstar Omega (BMG Labtech, Cary, USA), and samples (in technical triplicate) from non-treated and TGF-β1-treated LX-2 cells with a 260/280 ratio ≥ 1.9 were submitted for RNA sequencing.

### RNA sequencing pipeline

This work was performed by the Genomics Central Technology Unit (GCTU) of Queen’s University Belfast. RNA sequencing libraries were generated using an automated KAPA RNA HyperPrep kit with riboerase protocol (Roche, Basel, Switzerland) according to the manufacturer’s instructions on the Beckman FX^P^ robot (Beckman Coulter, Indianapolis, USA). Sequencing was performed on the Illumina Next-Seq 550 platform (Illumina, California, USA) using a 75 base-pair single-read flow cell. An average of 21,559,518 reads were obtained across all samples. Sequencing data was aligned to the human reference genome (assembly GRCh37, BioProject accession PRJNA31257) using the STAR aligner (version 2.7) [13] in Linux, and gene counts were calculated from the alignment data using HTSeq (version 0.11.1) [14]. Differential expression analysis was carried out on the data received from the GCTU using the DESeq2 (version 3.11) [15] analysis package in R (version 3.5.3) [16] with default settings applied.

### Pathway analysis

Pathway analysis was carried out on the gene expression data using Ingenuity Pathway Analysis (IPA) [17] software (Qiagen, Hilden, Germany). Genes were first mapped to the IPA knowledgebase, and the “core analysis” function was used to predict the canonical pathways that data set genes are associated with based on the gene fold change and false discovery rate (FDR) measurements. All analyses were carried out against the human knowledgebase with default settings applied. Changes in the activity of signalling pathways were quantified by the z-score, a value calculated through pathway analysis. The z-score is a directional measurement based on several factors, including the fold changes of the genes associated with a pathway, and the ratio of pathway genes present in the data set vs. those involved in the pathway overall [18]. A positive z-score indicates the pathway in question is more active compared to controls.

## Results

### TGF-β1 increased the formation of ACTA2 filaments in LX-2 cells

**Fig. 1:**
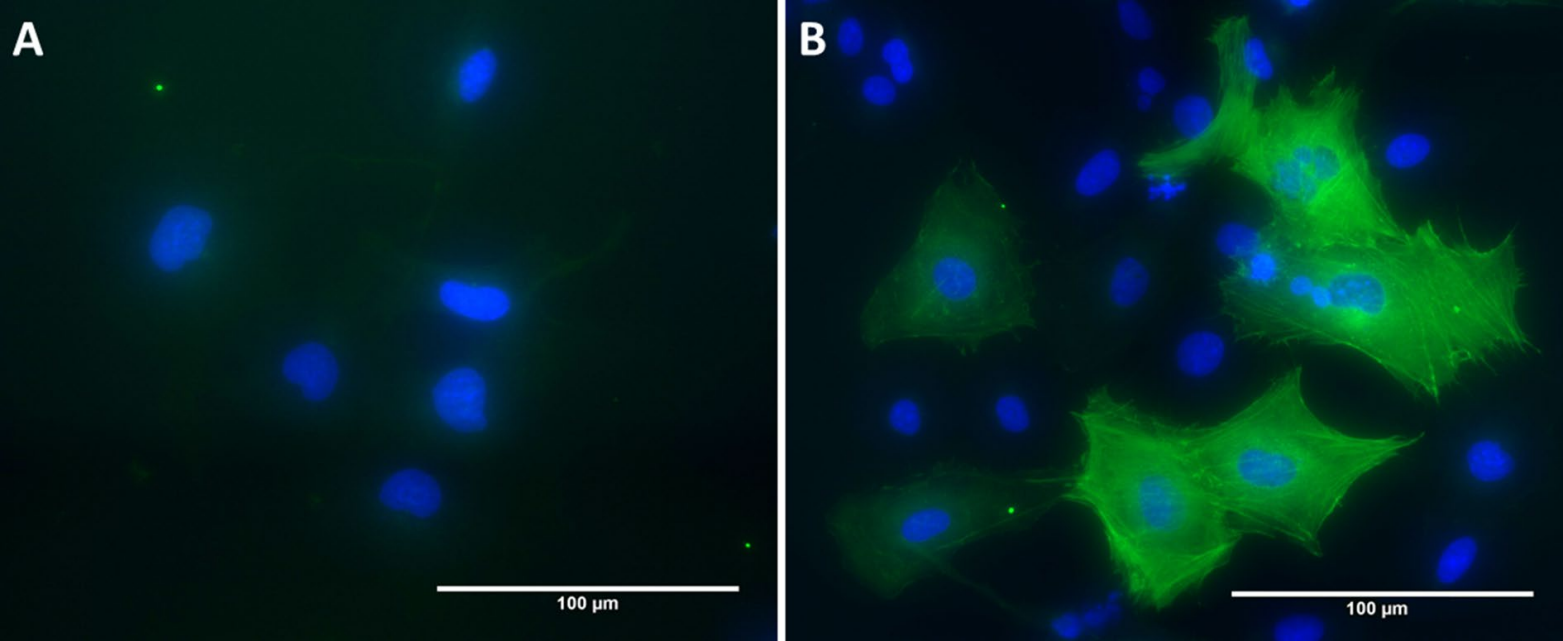
Representative fluorescent imaging of ACTA2 filaments in LX-2 cells. Cells shown stained with anti-ACTA and Alexa Fluor 488 secondary antibody. **A** non-treated or **B** treated with transforming growth factor-β1 (TGF-β1). As can be seen, TGF-β1 increased the appearance of organised ACTA2 filaments. Scale bar = 100 μm

The upregulated expression of ACTA2 and formation of organised ACTA2 filaments are common markers of myofibroblasts [5]. The presence of ACTA2 filaments within LX-2 cells was examined to confirm their activation following TGF-β1 exposure. Fluorescent microscopy (Fig. 1) confirmed that both the expression and the filament distribution of ACTA2 were clearly increased by TGF-β1 treatment.

### Differentially expressed genes in LX-2 cells following TGF-β1 treatment

**Fig. 2:**
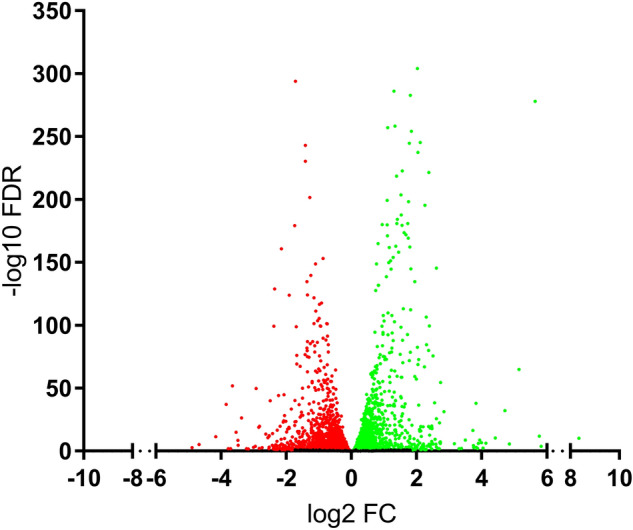
Differentially expressed genes in LX-2 cells following transforming growth factor-β1 (TGF-β1) treatment. The 17,821 differentially expressed genes detected in LX-2 cells following treatment with TGF-β1. The x-axis shows the gene log2 fold change (log2 FC) value and the y-axis shows the -log10 false discovery rate (− log10 FDR). Data points in green correspond with upregulated genes while points in red correspond with downregulated genes. Data points in black correspond with genes with non-significant changes in expression. The FDR cut-off for significance was < 0.1

The expression of 17,821 genes were detected in LX-2 cells. Of these genes, 5258 were observed to undergo statistically significant (FDR < 0.1) changes in expression following TGF-β1 treatment (2721 upregulated, 2537 downregulated). Figure 2 shows a volcano plot of the distribution of these genes. Tables 1 and 2 show the 25 most up- and downregulated genes detected in LX-2 cells following TGF-β1 treatment, respectively. The most upregulated genes included ISLR2 (fold change 324.03, FDR 6.06E-11) and KRT3 (fold change 56.49, FDR 1.71E-04). The most downregulated genes included SOX3 (fold change − 33.33, FDR 1.58E-03) and NR5A2 (fold change − 25.00, FDR 5.29E-06).

### Signalling pathways deregulated by TGF-β1 in LX-2 cells

**Fig. 3:**
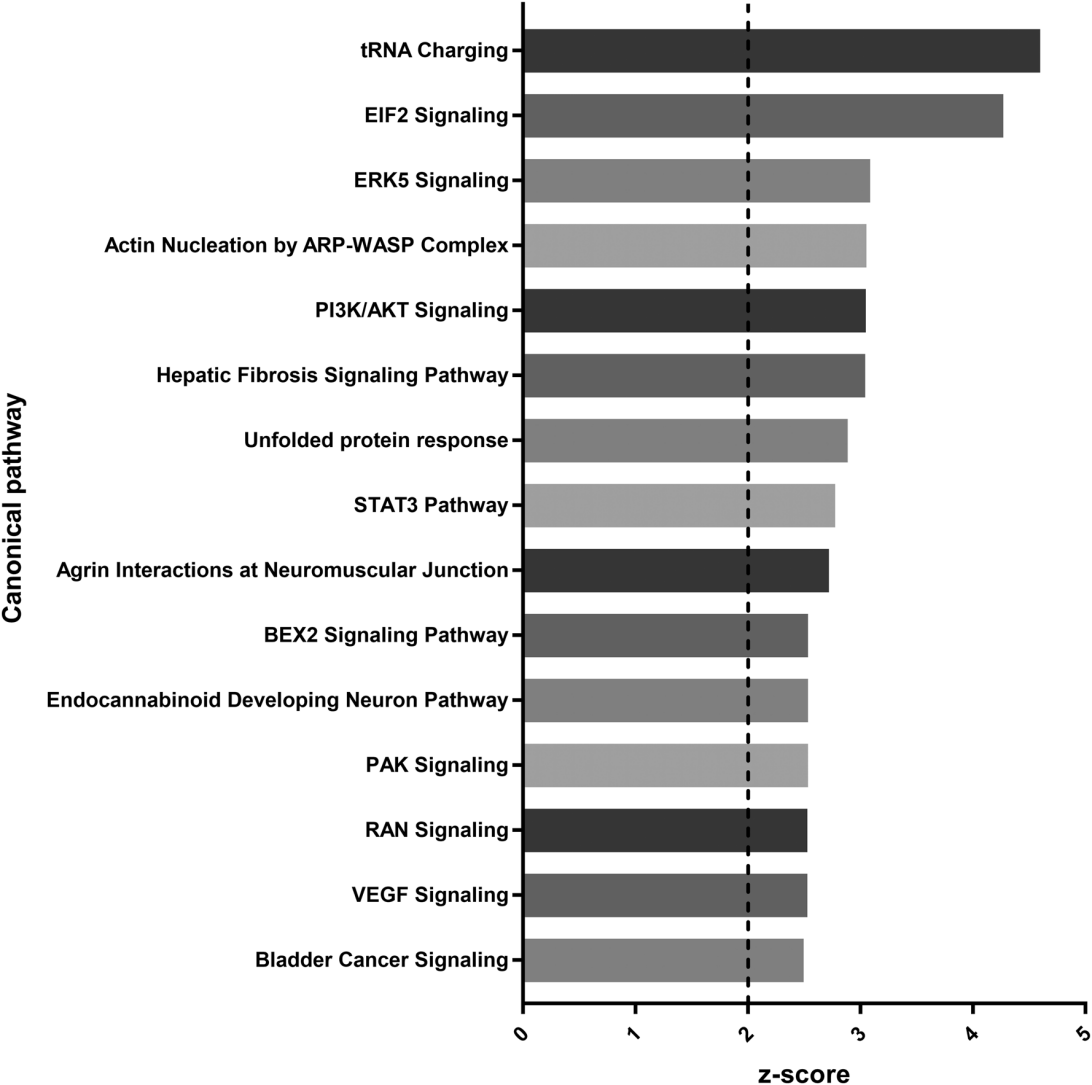
Signalling pathways upregulated by transforming growth factor-β1 (TGF-β1) in LX-2 cells. The top 15 signalling pathways predicted to be upregulated in LX-2 cells following TGF-β1 treatment (*p* value < 0.05, z-score > 2)

**Fig. 4:**
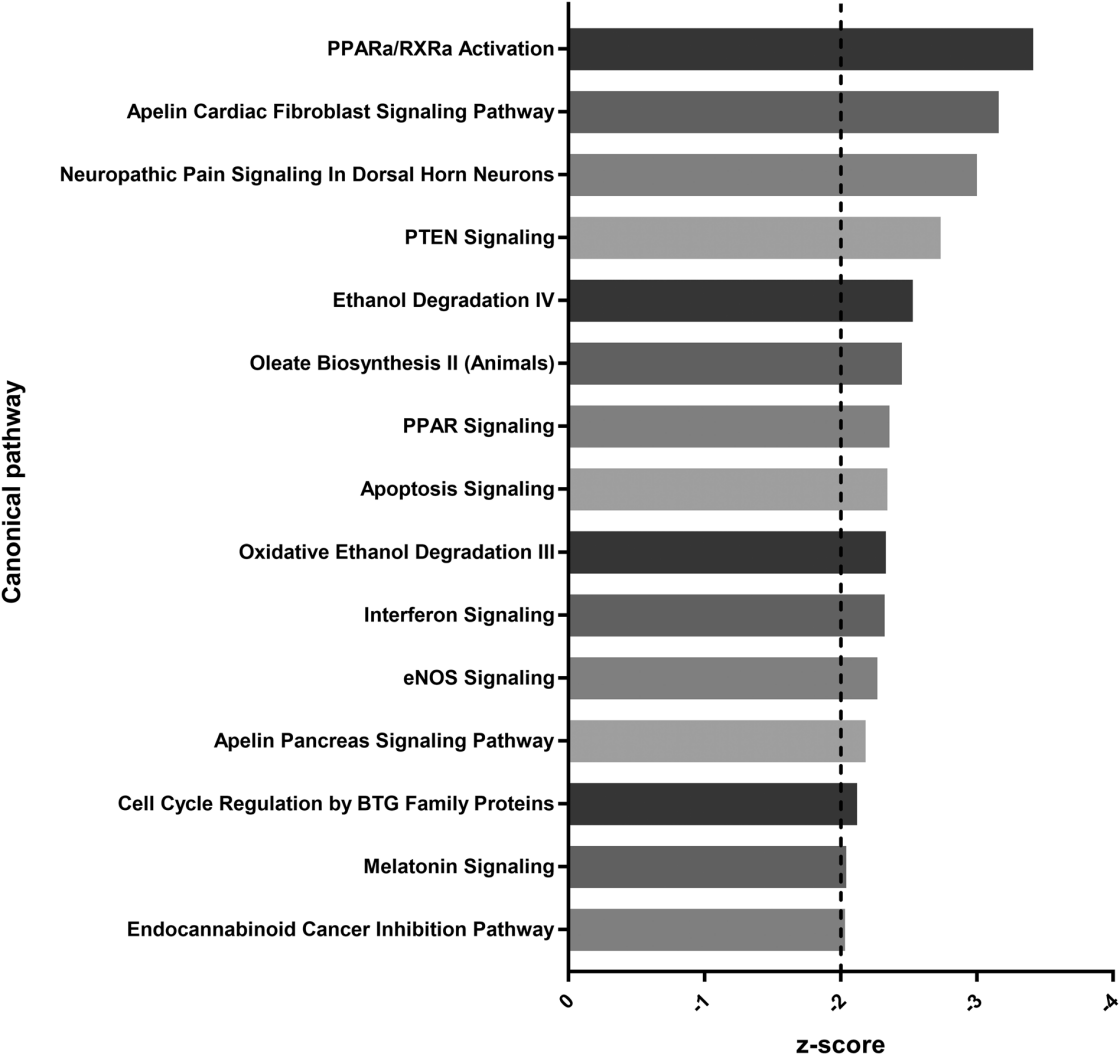
Signalling pathways downregulated by transforming growth factor-β1 (TGF-β1) in LX-2 cells. The top 15 signalling pathways predicted to be downregulated in LX-2 cells following TGF-β1 treatment (*p* value < 0.05, z-score < − 2)

Overall, the activity of 323 signalling pathways were predicted to be significantly (*p* value < 0.05) altered by TGF-β1 in LX-2 cells. The directional prediction of pathways with a z-score of between 2 and − 2 was assumed to be non-significant based on previous studies [18] and so these pathways were discounted. Figures 3 and 4 show the 15 most up- and downregulated pathways, respectively. The five most upregulated pathways included “tRNA charging” (z-score= 4.6), “EIF2 signalling” (z-score= 4.272), “ERK5 signalling” (z-score= 3.087), “actin nucleation by ARP-WASP complex” (z-score= 3.053) and “PI3K/AKT signalling” (z-score= 3.048). The five most downregulated pathways included “PPARα/RXRα activation” (z-score= − 3.414), “apelin cardiac fibroblast signalling pathway” (z-score= − 3.162), “neuropathic pain signalling in dorsal horn neurons” (z-score − 3), “PTEN signalling” (z-score − 2.734) and “ethanol degradation IV” (z-score= − 2.53).

## Discussion

Fibrosis is a pathology associated with many liver diseases, particularly chronic conditions, that can develop into cirrhosis, liver failure and death if left untreated [4]. Liver fibrogenesis often occurs when HSCs become activated following liver damage or disease and respond with the secretion of ECM proteins [19]. HSCs can activate in response to a range of stimuli, with one of the most common being TGF-β1 [9, 10]. TGF-β1 is a potent cytokine expressed throughout mammalian tissues, and is involved in a wide variety of key cellular processes [10]. Despite the physiological importance of TGF-β1 and its potent HSC activating ability, the specific responses the cytokine induces in HSCs have yet to be fully characterised due to the complexity and far reaching nature of TGF-β1 signalling [1].

Several studies have explored HSC activation at the transcriptomic level using various methods and cell lines [20–25]. The first such study utilised microarray analysis to investigate the effects of culture-induced activation (where HSCs activate over time on tissue culture plastic) on gene expression in LI90 cells, another immortalised human HSC line, when cultured on Matrigel [24]. This study identified 3350 differentially expressed genes and led to the identification of myocardin as an activator of HSCs [24]. A second study used RNA sequencing of primary human foetal HSCs exposed to TGF-β1 to identify differentially expressed long non-coding RNAs (lncRNAs) [21]. This study found that TGF-β1 influences the expression of 381 lncRNAs in human foetal HSCs [21]. Another RNA sequencing study investigated the differences in gene expression between quiescent and culture-activated primary human HSCs, with valproic acid used to maintain quiescence [20]. Overall, the differential expression of 5,449 genes were detected and three genes which regulate the expression of connective tissue growth factor, fibroblast growth factor 2 and netrin 4, each associated with HSC activation and liver fibrosis, were identified [20]. RNA sequencing has also been applied to assaying the transcriptomic effects of anti-fibrotic molecules on HSCs with the aim of identifying potential therapeutics for liver fibrosis [26].

As noted by Gerhard et al., the aim of these studies can be put simply as characterising the changes in gene expression that occur in HSCs during activation, and yet the findings show a large amount of variation in both the identity and number of differentially expressed genes [25]. It is clear that the methods used to provoke HSC quiescence or activation, and detect gene expression, as well as the specific cell lines assayed, have a strong impact on the final results [25].

A summary of the effects of TGF-β1 on the genes and signalling pathways discussed below can be found in Table 3.

### Genes deregulated by TGF-β1 in LX-2 cells

Several genes described in Table 1 have known roles in promoting HSC activation and liver fibrosis downstream of TGF-β1, including EGR2, FAP, FN1, HES1 and NOX4 [27–31]. While the function of these genes in relation to liver fibrosis is known, their highly upregulated status in this context may indicate that they are particularly significant mediators of early HSC activation or TGF-β1 signalling, and therefore worthy of more attention as potential markers for activating HSCs or fibrogenesis.

Other genes were identified in Table 1 that do not have clearly reported roles in HSCs. These genes have instead been associated with either the activity of fibroblasts or fibrogenesis in other tissues, including FOXS1, TGFβI, PI16, VIP and PRG4. FOXS1 promotes the activation of primary human skin fibroblasts [32], while TGFβI has been shown to interact with ECM proteins, including collagen type 1 (COL1), to inhibit the cell-ECM adhesion of skin and scleral fibroblasts [33]. The upregulation of these genes seen here may indicate that FOXS1 also promotes HSC activation downstream of TGF-β1, while TGFβI is likely involved in facilitating the migration of early activating HSCs from the space of Dissé. The overexpression of PI16 has been shown to reduce the proliferation of, and expression of COL1 in, murine cardiac fibroblasts [34]. Similarly, the reduced expression of VIP correlates with progressive cardiac fibrosis in murine models, which can be reversed by VIP overexpression [35]. PRG4 is associated with protection functions in the connective tissues and reduced fibroblast activation in the synovial tissue [36]. Assuming these genes carry out similar functions in HSCs, their upregulation by TGF-β1 is indicative of negative regulation of HSC activation, likely as a means of controlling fibrosis progression.

Several downregulated genes whose function likely influences HSC activity were identified in Table 2, including CIITA, SERPINB2 and PSG1. The upregulation of CIITA results in the increased expression of major histocompatibility complex class II (MHCII) genes [37], which have been shown to reduce HSC collagen expression and fibrotic potential during schistosomiasis infection [38]. It can therefore be assumed that the downregulated CIITA expression seen here would increase HSC collagen expression and contribution to fibrosis [38]. A deficiency of SERPINB2 in the livers of murine models of the helminth *Schistosoma japonicum* infection results in a reduction in the deposition of collagen within the egg-induced granuloma [39]. Given the role of HSCs within the granuloma, it is highly likely that SERPINB2 deficiency reduces HSC activity to bring about this effect and, if so, would implicate SERPINB2 as a promoter of HSC activity. PSG1 has been shown to stimulate the release of active TGF-β1 protein *in vitro* [40], and therefore its reduced expression in this context would inhibit TGF-β1 signalling and subsequent HSC activation.

The expression of COL17A1 and COL4A6 were also downregulated, despite COL4 having been shown previously to be upregulated in HSCs following TGF-β1 exposure [41]. One previous study has shown that COL17 and COL4 interact together in skin and oral keratinocytes to assist cell-ECM adhesion [42]. COL4 has been identified as an ECM component in the space of Dissé, the storage site of quiescent HSCs, while COL17 is a transmembrane collagen that interacts with both extra- and intracellular structural components to facilitate cell linkage to the epithelium. Given that activating HSCs must migrate from the space of Dissé towards the provoking stimuli, it is possible that the expression of these collagens, perhaps working in tandem with TGFβI, might be initially downregulated in order to allow the cell to disengage from the anchoring ECM in the space of Dissé, and thus allow migration.

**Table 1:** Genes upregulated by transforming growth factor-β1 (TGF-β1) in LX-2 cells

Gene ID	Gene name	Fold change	FDR
ISLR2	Immunoglobulin superfamily cont. leucine rich repeat 2	324.03	6.06E-11
KRT3	Keratin 3	56.49	1.71E-04
FOXS1	Forkhead box S1	54.19	1.18E-12
PMEPA1	Prostate transmembrane protein, androgen induced 1	49.87	8.20E-279
EGR2	Early growth response 2	35.26	1.40E-65
SYN1	Synapsin I	28.84	2.16E-06
FAP	Fibroblast activation protein	26.17	5.84E-33
SCN7A	Sodium voltage-gated channel alpha subunit 7	21.26	3.76E-11
STRA6	Signalling receptor and transporter of retinol	15.89	1.47E-07
PI16	Peptidase inhibitor 16	14.83	7.01E-07
VIP	Vasoactive intestinal peptide	14.62	8.14E-03
NOX4	NADPH oxidase 4	14.22	1.08E-03
LRRC15	Leucine rich repeat containing protein 15	14.12	9.03E-39
PRG4	Proteoglycan 4	13.45	1.31E-03
GAL	Galanin and GMAP prepropeptide	13.36	3.27E-05
DSP	Desmoplakin	11.71	0.00E+00*
UNC5B	Unc-5 netrin receptor B	11.31	1.78E-09
GUCY1A3	Guanylate cyclase soluble subunit alpha-3	10.34	6.82E-03
KANK4	KN motif and ankyrin repeat domains 4	9.85	1.18E-02
TGFBI	Transforming growth factor beta induced	9.58	0.00E+00*
FN1	Fibronectin 1	8.06	0.00E+00*
SLAMF8	SLAM family member 8	7.16	3.95E-32
HES1	Hes family BHLH transcription factor 1	6.68	2.24E-55
MICALCL	MICAL C-terminal like	6.63	7.93E-07
CCL7	Chemokine (C-C motif) ligand 7	6.59	5.00E-08

* FDR value of 0.0 owing to a limitation in R software that returns values lower than 2.2E-308 as 0.0. These genes were assigned a FDR value of 1E-308 for pathway analysis

**Table 2:** Genes downregulated by transforming growth factor-β1 (TGF-β1) in LX-2 cells

Gene ID	Gene name	Fold change	FDR
SOX3	SRY-box transcription factor 3	− 33.33	1.58E-03
NR5A2	Nuclear receptor subfamily 5 group A member 2	− 25.00	5.29E-06
LRRC7	Leucine rich repeat containing protein 7	− 16.67	2.56E-12
SERPINB2	Serpin family B member 2	− 12.50	1.10E-52
SEMA3B	Semaphorin-3B	− 11.11	1.12E-15
COL17A1	Collagen type 17 α1 chain	− 11.11	2.76E-09
VCAM1	Vascular cell adhesion molecule 1	− 11.11	1.83E-05
EVI2B	Ecotropic viral integration site 2B	− 10.00	4.53E-27
ZNF665	Zinc finger protein 665	− 9.09	1.22E-02
PSG1	Pregnancy specific β-1-glycoprotein 1	− 9.09	1.41E-02
PTPRC	Protein tyrosine phosphatase receptor type C	− 7.69	1.25E-04
SEMA3A	Semaphorin-3 A	− 7.69	1.84E-50
SLC27A2	Solute carrier family 27 member 2	− 7.14	9.88E-20
EVI2A	Ecotropic viral integration site 2 A	− 7.14	1.32E-20
PTPRN2	Protein tyrosine phosphatase receptor type N2	− 6.67	1.65E-03
MSTN	Myostatin	− 5.88	2.40E-11
GRIA1	Glutamate ionotropic receptor AMPA type subunit 1	− 5.88	5.08E-04
PLEKHG4	Pleckstrin homology and RhoGEF domain containing G4	− 5.88	5.91E-13
COL4A6	Collagen type 4 α6 chain	− 5.88	1.37E-12
PPL	Periplakin	− 5.56	6.90E-41
ADRA1B	Alpha-1B adrenergic receptor	− 5.26	1.85E-05
CHRM2	Cholinergic receptor muscarinic 2	− 5.26	1.12E-14
CIITA	Class II major histocompatibility complex transactivator	− 5.26	1.43E-02
GALNT5	Polypeptide N-acetylgalactosaminyltransferase 5	− 5.26	4.83E-04
GRIN2A	Glutamate ionotropic receptor NMDA type subunit 2 A	− 5.26	5.18E-100

### Signalling pathways upregulated by TGF-β1 in LX-2 cells

The most strongly upregulated signalling pathway in Fig. 3 was that of transfer (t)-RNA charging, a pathway involved with protein translation. Increased tRNA charging activity is synonymous with the increased level of protein synthesis that occurs in HSCs during, and following, activation [5]. Similarly, eukaryotic translation initiation factor 2 (EIF2) signalling is important in the initiation of protein synthesis in eukaryotic cells [5]. However, one study has reported that a component of the *S. mansoni* EIF2 signalling pathway, the subunit EIF2α, can interact with the TGF-β receptors TGFβRI and TGFβRII to inhibit TGF-β signalling [43]. The nature of the enhanced EIF2 signalling in aHSCs following TGF-β1 exposure could therefore also double as a negative regulator of TGF-β1 responses.

Several pathways in Fig. 3, including ERK5, PI3K/AKT and STAT3 signalling, represent signalling cascades downstream of TGF-β1 that are capable of driving HSC activation [44]. TGF-β1 carries out physiological functions by inducing cellular gene expression, and the SMAD family of transcriptional regulators are generally responsible for transducing signals from TGF-β ligands to the cell nucleus [10]. The absence of such signalling from the data could suggest that, while highly active immediately following TGF-β1 exposure, by the 24-hour timepoint SMAD signalling gives way to these alternative, SMAD-independent pathways. This likely occurs to balance preventing excessive HSC activation whilst simultaneously inducing pro-fibrotic gene expression in aHSCs.

The assembly of organised ACTA2 filaments is a strong marker of myofibroblasts [5] (see Fig. 1). These filaments carry out several functions in aHSCs, including supporting the expanding cell cytoplasm, facilitating cell motility and acting as a method of attaching to, and signalling between, the ECM and other cells [5, 45]. Therefore, it is unsurprising that the activity of the actin nucleation by ARP-WASP complex was upregulated by TGF-β1 exposure.

### Signalling pathways downregulated by TGF-β1 in LX-2 cells

The most strongly downregulated signalling pathway in Fig. 4 was that of PPARα/RXRα activation, and PPAR signalling was also found to be downregulated. Quiescent HSCs take up and store vitamin A (retinol) within lipid droplets [5] following its metabolism into lipid-soluble derivatives [46]. HSCs regulate the expression of genes involved in fatty acid uptake and metabolism via peroxisome proliferator-activated receptors (PPARs) and the retinoid X receptor (RXR), which heterodimerise together to act as a transcription factor for these genes [46]. Upon activation, HSCs lose the ability to store vitamin A and, as such, display reduced retinol-related signalling [47]. Studies have shown that the expression of both PPAR-γ, a relative of PPAR-α, and RXR are reduced in aHSCs [47, 48]. Furthermore, agonism of PPAR-γ signalling in aHSCs has been shown to suppress the expression of ACTA2 and collagen type 1α1 (COL1A1), and to facilitate aHSC reversion back into a quiescent state [48]. As such, the downregulated activity of the PPARα/RXRα activation and PPAR signalling pathways following TGF-β1 exposure was expected.

Apelin is an endogenous ligand of the G protein-coupled APJ receptor. In the liver, apelin signalling is strongly associated with fibrosis; several studies have highlighted how components of the apelin signalling pathway induce the expression of pro-fibrotic genes in LX-2 cells, including COL1, ACTA2 and platelet-derived growth factor receptor-β (PDGFRβ) [49]. Furthermore, the inhibition of apelin signalling has been shown to reduce the intensity and burden of liver fibrosis in murine models [50]. Paradoxically however, other studies have linked apelin signalling with the inhibition of TGF-β1 responses; one study has shown that apelin inhibits the TGF-β1-induced activation of SMAD proteins and subsequent upregulation of ACTA2, COL1 and FN1 expression in epithelial cells [51], while another described how apelin inhibits the TGF-β1-induced upregulation of ACTA2 and COL1A1 expression in cardiac fibroblasts [52]. These findings highlight the tissue-specific nature of apelin signalling and could indicate an interesting situation in HSCs whereby apelin increases fibrotic gene expression whilst simultaneously inhibiting TGF-β1 signalling.

Phosphatase and tensin homolog (PTEN) is a tumour suppressor protein that regulates cell cycle progression. Several studies have shown that PTEN signalling inhibits HSC activation; one study demonstrated that the downregulation of miR-181b, an inhibitor of PTEN expression, results in the suppression of HSC activation as determined by reduced ACTA2 expression and collagen deposition [53]. Another study showed that PTEN-deficient mice develop progressive liver fibrosis characterised by the increased expression of ACTA2, COL1 and tissue inhibitor of matrix metalloproteinase (TIMP)-1 [54]. HSCs isolated from these PTEN-deficient mice displayed higher levels of activation on average compared to HSCs in wild type mice [54]. Similarly, one final study has described how the overexpression of PTEN in rat HSCs prevents the morphological changes associated with activation, and reduces the expression of ACTA2 and COL1A1 [55]. Taken together, PTEN signalling is a strong negative regulator of HSC activation.

Ethanol and its metabolites have been shown to promote HSC activation through several mechanisms [56]. Given the strong activating influence of ethanol and acetaldehyde in HSCs, it is unusual that TGF-β1 exposure would downregulate the activity of several ethanol degradation pathways. HSCs express enzymes involved in ethanol degradation; however, it is possible that activated HSCs may inhibit the expression of these enzymes in an attempt to regulate ethanol-induced activation and fibrosis as a protective mechanism.

**Table 3:** Summary of the deregulating effects of transforming growth factor-β1 (TGF-β1) on genes and pathways in LX-2 cells

Gene	Effect of TGF-β1	Phenotype	Pathway	Effect of TGF-β1	Phenotype
CIITA	Downregulated	Not characterised	Actin nucleation by ARP-WASP complex	Upregulated	Activated
COL17α1	Downregulated	Not characterised	Apelin signalling	Downregulated	Activated
COL4α6	Downregulated	Not characterised	EIF2 signalling	Upregulated	Activated
EGR2	Upregulated	Activated	ERK5 signalling	Upregulated	Activated
FAP	Upregulated	Activated	Ethanol degradation	Downregulated	Activated
FN1	Upregulated	Activated	PI3K/AKT signalling	Upregulated	Activated
FOXS1	Upregulated	Not characterised	PPAR signalling	Downregulated	Quiescent
HES1	Upregulated	Activated	PPARα/RXRα activation	Downregulated	Quiescent
NOX4	Upregulated	Activated	PTEN signalling	Downregulated	Quiescent
PI16	Upregulated	Not characterised	STAT3 signalling	Upregulated	Activated
PRG4	Upregulated	Not characterised	tRNA charging	Upregulated	Activated
PSG1	Downregulated	Not characterised	Unfolded protein response	Upregulated	No effect
SEMA3A	Downregulated	Not characterised			
SERPINB2	Downregulated	Not characterised			
TGFβI	Upregulated	Not characterised			
VCAM1	Downregulated	Not characterised			
VIP	Upregulated	Not characterised			

The deregulating effects of TGF-β1 on the genes and signalling pathways discussed above, and the result of this deregulation on the activation status of hepatic stellate cells (HSCs). Genes whose role within HSCs is unknown are listed as “not characterised”

## Conclusions

Our findings highlight the most strongly deregulated genes and signalling pathways in LX-2 cells in the early response to TGF-β1. While several of the genes identified are known influencers of HSC activation, many have no thoroughly characterised role in HSCs and their relevance to fibrosis was inferred from activities in other cell types and tissues. Characterising the role of these genes within HSCs could be a useful point for further study in order to identify any genes with novel roles in HSC activation.

As expected, TGF-β1 influenced signalling pathway activity in a direction that favoured HSC activation. Broadly speaking, most of the pathways upregulated by TGF-β1 can be categorised according to their involvement in either SMAD-independent transcriptional regulation, protein translation regulation, or regulation of the actin cytoskeleton. Conversely, the pathways downregulated by TGF-β1 cover a broader range of signalling processes that are harder to categorise. While we did not identify any novel fibrosis-associated processes occurring within LX-2 cells, the identification of the specific pathways most involved in the early LX-2 cell response to TGF-β1 is useful for the improved understanding of the impacts of TGF-β1 signalling in HSCs.

## Data Availability

The dataset generated during the current study is available in the National Center for Biotechnology Information (NCBI) repository (BioProject PRJNA680982, available at https://www.ncbi.nlm.nih.gov/bioproject/PRJNA680982).

## Abbreviations

ACTA2: α-smooth muscle actin
aHSC: activated hepatic stellate cell, myofibroblast
ALD: alcoholic liver disease
CIITA: class II major histocompatibility complex transactivator
COL1A1: collagen type 1 chain 1
ECM: extracellular matrix
EGR2: Early growth response 2
EIF2: eukaryotic initiation factor 2
FAP: fibroblast activation protein
FDR: false discovery rate
FN1: fibronectin 1
FOXS1: forkhead box S1
HCV: hepatitis C virus
HES1: hes family BHLH transcription factor 1
HSC: hepatic stellate cell.
ISLR2: immunoglobulin superfamily cont. leucine rich repeat 2
KRT3: keratin 3
MHCII: major histocompatibility complex class II
NAFLD: non-alcoholic fatty liver disease
NASH: non-alcoholic steatohepatitis.
NOX4: NADPH oxidase 4
NR5A2: nuclear receptor subfamily 5 group A member 2
PI16: peptidase inhibitor 16
PPAR: peroxisome proliferator-activated receptor
PRG4: proteoglycan 4
PSG1: pregnancy specific β-1-glycoprotein 1
PTEN: phosphatase and tensin homolog
RXR: retinoid X receptor
SERPINB2: serpin family B member 2
SOX3: SRY-box transcription factor 3
TGF-β1: transforming growth factor-β1
TGFβI: transforming growth factor-β induced
TGFβR: transforming growth factor-β receptor
VIP: vasoactive intestinal peptide
